# Binary Type-II Heterojunction K_7_HNb_6_O_19_/g-C_3_N_4_: An Effective Photocatalyst for Hydrogen Evolution without a Co-Catalyst

**DOI:** 10.3390/nano12050849

**Published:** 2022-03-02

**Authors:** Qi Song, Shiliang Heng, Wenbin Wang, Huili Guo, Haiyan Li, Dongbin Dang

**Affiliations:** Henan Key Laboratory of Polyoxometalate Chemistry, College of Chemistry and Chemical Engineering, Henan University, Kaifeng 475004, China; songqi1666@163.com (Q.S.); hengshiliangecnu@163.com (S.H.); wwenbin2022@163.com (W.W.); ghlhenu@163.com (H.G.); lihaiyan@henu.edu.cn (H.L.)

**Keywords:** PONb-based photocatalyst, g-C_3_N_4_, type-II heterojunction, photocatalytic hydrogen evolution

## Abstract

The binary type-II heterojunction photocatalyst containing g-C_3_N_4_ and polyoxoniobate (PONb, K_7_HNb_6_O_19_) with excellent H_2_ production activity was synthesized by decorating via a facile hydrothermal method for the first time. The as-fabricated Nb–CN-0.4 composite displayed a maximum hydrogen evolution rate of 359.89 µmol g^−1^ h^−1^ without a co-catalyst under the irradiation of a 300 W Xenon Lamp, which is the highest among those of the binary PONb-based photocatalytic materials reported. The photophysical and photochemistry analyses indicated that the hydrogen evolution performance could be attributed to the formation of a type-II heterojunction, which could not only accelerate the transfer of photoinduced interfacial charges, but also effectively inhibit the recombination of electrons and holes. This work could provide a useful reference to develop an inexpensive and efficient photocatalytic system based on PONb towards H_2_ production.

## 1. Introduction

A looming global energy shortage makes it increasingly urgent to develop environmentally friendly green chemical technology for energy production to ensure the sustainable development of human society [1,2,3]. In recent decades, much effort has been devoted to developing the production of hydrogen, not merely because it is an important chemical raw material; more importantly, hydrogen energy is recognized as the most ideal and promising clean energy of the future [4]. Due to its environmental friendliness, efficient cost performance and non-polluting nature, photocatalytic technology has been widely used to produce such green energy [5]. Photocatalysis water splitting exhibits a great potential in hydrogen production and is expected to achieve industrialization [6,7]. However, the development of low-cost and highly efficient photocatalysts for hydrogen production is still a major challenge for practical application.

Polyoxometalate (POM) is a kind of metal oxide cluster based mainly on Mo, W, V, Nb and Ta elements [8], which have been widely studied due to their promising applications in fields such as water oxidation, hydrogen evolution, carbon dioxide reduction, and nitrogen reduction reaction [9]. As a special subclass of POMs with the unique electronic characteristics of high charge density and rapid electron transfer rate, polyoxoniobates (PONbs) have been employed to assemble photocatalytic composites with other semiconductor species [10]. It is worth mentioning that the Lindqvist type K_7_HNb_6_O_19_ is a favorite candidate to be selected to be as a model of PONbs to investigate the construction of PONb-based hydrogen-evolution photocatalysts, for its easy preparation and high stability [11]. However, immobilization and reusability are a tremendous challenge for such materials because of the water solubility, highly alkalinity, and confined working pH region of the components of K_7_HNb_6_O_19_ [12,13].

Graphitic carbon nitride (g-C_3_N_4_, CN) is reported to be a promising photocatalyst for hydrogen evolution because of its appropriate band gap and energy structure being capable of performing water reduction reaction [14,15]. However, the photocatalytic hydrogen production activity of pure g-C_3_N_4_ is confined by the rapid electron-hole recombination and tardy charge mobility, leading to the low photocatalytic activity [16,17]. A large number of researchers have shown that the photocatalytic activity of g-C_3_N_4_ can be enhanced by morphology regulation, element doping, dye sensitization, heterojunction construction, various noble metals loading (Pt, Au), and other methods [18,19,20,21,22]. Among them, two strategies of loading noble metals and constructing heterojunctions have been employed widely as effective ways to boost the catalytic property of g-C_3_N_4_ [23]. Nevertheless, constructing heterojunctions by combining with other semiconductors is a preferred method to fabricate g-C_3_N_4_-based photocatalysts on account of the high scarcity and cost of noble metals [24]. It was proven that the combination of g-C_3_N_4_ and POMs is an effective strategy to obtain heterojunctions with improved photoactivity, which not only gives them a large specific surface and a narrowed band gap, but also could accelerate the photoinduced interfacial charge transfer and inhibit effectively the recombination of the electrons and holes in the process of photocatalysis. For example, a series of type II heterojunction composites (POMs/C_3_N_4_) were reported based on Keggin-type polyoxoanions SiW_12_O_40_^4−^, PW_12_O_40_^3−^ and PMo_12_O_40_^3−^ [25]. Up to now, the reported POM-based g-C_3_N_4_ hybrid materials such as Co_4_PW_9_O_34_/g-C_3_N_4_, FePW_4_O_24_/g-C_3_N_4_ and PMo_10_V_2_O_40_/g-C_3_N_4_ have mainly been based on acidic POMs [26,27,28]. As for the highly basic PONbs, there have only been a few examples reported, such as Mg_3_Al–LDH–Nb_6__,_ CdS/K_7_HNb_6_O_19_/NiS, K_8_Nb_6_O_19_/g-C_3_N_4_ and K_4_Nb_6_O_17_/g-C_3_N_4_ [12,29,30,31]. In this work, a series of K_7_HNb_6_O_19_/g-C_3_N_4_ photocatalysts were fabricated via a facile hydrothermal method, which was used for effective H_2_ generation without any co-catalyst. To the best of our knowledge, this is the first study constructing a binary K_7_HNb_6_O_19_/g-C_3_N_4_ type-II heterojunction photocatalyst, and it possesses outstanding photocatalytic H_2_ evolution properties under ultraviolet light driving.

## 2. Experimental Section

### 2.1. Chemicals and Reagents

All chemicals were reagent grade and used without further purification. Niobium pentoxide (Nb_2_O_5_, AR), methyl alcohol (MeOH, AR), potassium hydroxide (KOH, AR), sodium sulfate (Na_2_SO_4_, AR), ethanol (EtOH, AR) and urea (CO(NH_2_)_2_, AR) were purchased from Sinopharm Chemical Reagent Co., Ltd. (Shanghai, China).

### 2.2. Synthesis of K_7_HNb_6_O_19_·13H_2_O

K_7_HNb_6_O_19_·13H_2_O was synthesized according to the method reported in the literature [32]. Typically, 13.3 g Nb_2_O_5_ and 26 g KOH were added into the nickel crucible and mixed evenly. The resultant mixture was heated in a muffle furnace at 480 °C for 50 min; then the product was collected and dissolved in 100 mL boiling water after it was cooled to room temperature. When the volume of the solution reached about 50 mL by evaporating water, the heating was stopped. The white solids were collected and dried in vacuum.

### 2.3. Synthesis of g-C_3_N_4_

The g-C_3_N_4_ was prepared through the direct calcination of urea according to the methods reported in the literature [33]. Typically, urea was placed evenly in a covered crucible and heated at 550 °C for 3 h in a tube furnace, with a heating rate of 10 °C min^−1^. The resultant product was taken out when the temperature cooled to about 30 °C and then ground for use.

### 2.4. Synthesis of Nb–CN-X Composites

**Figure 1:**
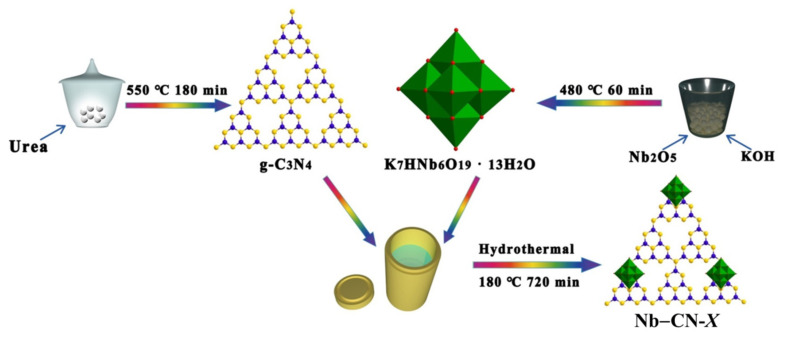
Schematic illustration of the fabrication of Nb–CN-*X* composites.

Nb–CN-*X* composite photocatalysts were prepared according to the following process, where *X* refers to the mass of g-C_3_N_4_ added based on 1 g K_7_HNb_6_O_19_ as a benchmark; *X* = 0.2, 0.4, 0.6, 0.8. Typically, taking Nb–CN-0.4 as an example, as-obtained 0.4 g g-C_3_N_4_ was added to a solution containing 1 g Linquist type K_7_HNb_6_O_19_ and 15 mL deionized water and stirred for 3 h to form yellow mixed suspension, and then it was transferred to a 25 mL Teflon-lined reactor and heated at 180 °C for 12 h. The resulting precipitate was collected and washed separately with high-purity water and ethanol three times and then dried in a vacuum oven at 60 °C for 12 h to obtain the white photocatalyst Nb–CN-0.4 composite. Similarly, other composites were also synthesized by adjusting the amount of g-C_3_N_4_ (*X* = 0.2, 0.6, 0.8) according to the same process. The preparation process is shown in Figure 1. The detailed characterization methods and analytical methods are described in the Supporting Information.

## 3. Results and Discussion

### 3.1. Morphology and Structure Analysis

**Figure 2:**
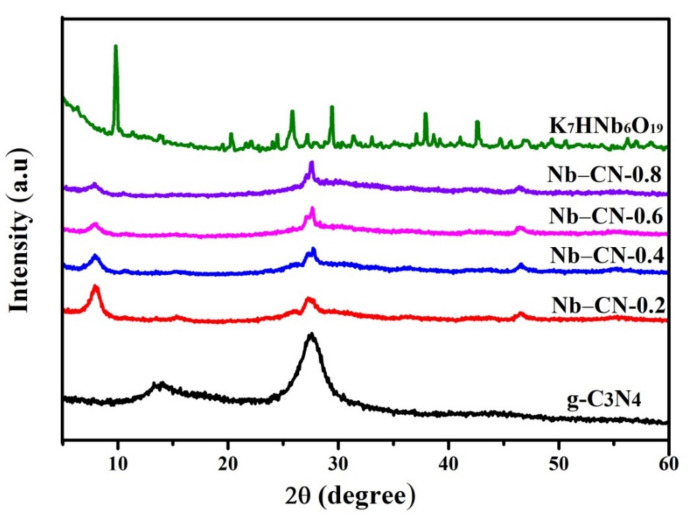
XRD patterns of g-C_3_N_4_, K_7_HNb_6_O_19_ and Nb–CN-*X* composites (*X* = 0.2, 0.4, 0.6, 0.8).

The crystalline phase of the as-prepared K_7_HNb_6_O_19_, g-C_3_N_4_ and Nb–CN-*X* composites were characterized by X-ray diffraction (XRD). As shown in Figure 2, the diffraction peaks of pure g-C_3_N_4_ are 13.3° and 27.5°, while the original K_7_HNb_6_O_19_ has sharp diffraction peaks at 9.8°, 26° and 48°, which is consistent with the reported results [30,34]. There are three distinct characteristic peaks located at the positions of 8.8°, 27.5° and 46° for Nb–CN-*X* composites. Among them, the peak at 27.5° matches with the (002) crystal plane of g-C_3_N_4_, indicating that g-C_3_N_4_ has been successfully introduced. However, the diffraction peak corresponding to the (100) crystal plane of g-C_3_N_4_ at 13.3° cannot be observed clearly in the Nb–CN-*X* composites with the introduction of K_7_HNb_6_O_19_, which may be due to the low content of g-C_3_N_4_. Concurrently, the sharp XRD pattern signals from the polyoxoniobate are not visible in the binary composites, while two new wide peaks at 8.8° and 46° are observed, which could be attributed to the peaks of K_7_HNb_6_O_19_ shifted from 9.8° and 48°. This phenomenon might be caused by the coupling between the two components K_7_HNb_6_O_19_ and g-C_3_N_4_ in the composites [35]. In addition, with the increasing of the content of g-C_3_N_4_ in the Nb–CN-*X* composites, the peak intensity of K_7_HNb_6_O_19_ decreased little by little, while that of g-C_3_N_4_ increased gradually, showing the successful composite of the two components in Nb–CN-*X*.

**Figure 3:**
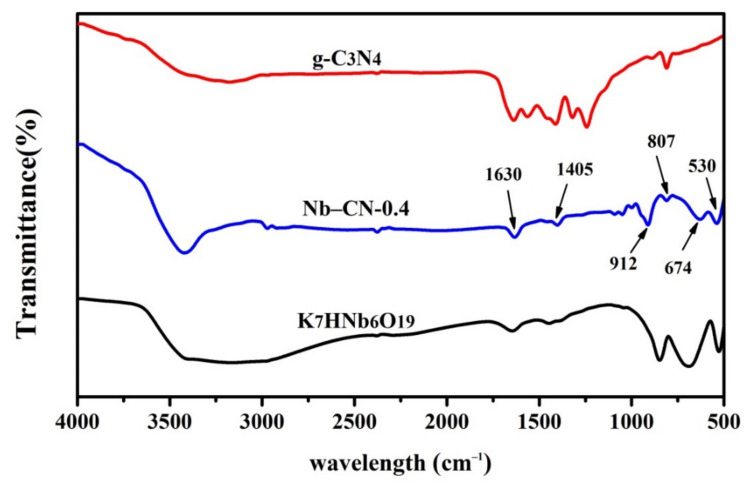
FT-IR spectra of pure g-C_3_N_4_, K_7_HNb_6_O_19_ and Nb–CN-0.4 composite.

To give the further information of the composite photocatalysts, the FT-IR spectra of the K_7_HNb_6_O_19_, g-C_3_N_4_ and Nb–CN-0.4 composite were carried out as representatives. As shown in Figure 3, for Nb–CN-0.4 composite, the three vibrational absorption peaks observed at 853, 674 and 530 cm^−1^ belonged to the tensile vibration of Nb–O and Nb–O–Nb of K_7_HNb_6_O_19_ [24,36]. The characteristic peaks of g-C_3_N_4_ in Nb–CN-0.4 appeared at different wavebands in the absorption region. The wide peak between 3000 and 3600 cm^−1^ was caused by the stretching vibration of N–H and O–H of the physical absorption water molecules [37]. The peaks at 1630 cm^−1^ are attributed to the stretching vibration of C=N in g-C_3_N_4_, while the peaks at 1405, 1321, 1246 and 807 cm^−1^ belonged to a typical C–N heterocyclic stretching vibration for triazinyl units [23,38]. Combined with the results of XRD and FT-IR, the conclusion can be drawn that under hydrothermal conditions, K_7_HNb_6_O_19_ was successfully embedded in g-C_3_N_4_, rather than simply mixed, to form the final hybrid material Nb–CN-0.4 composite.

**Figure 4:**
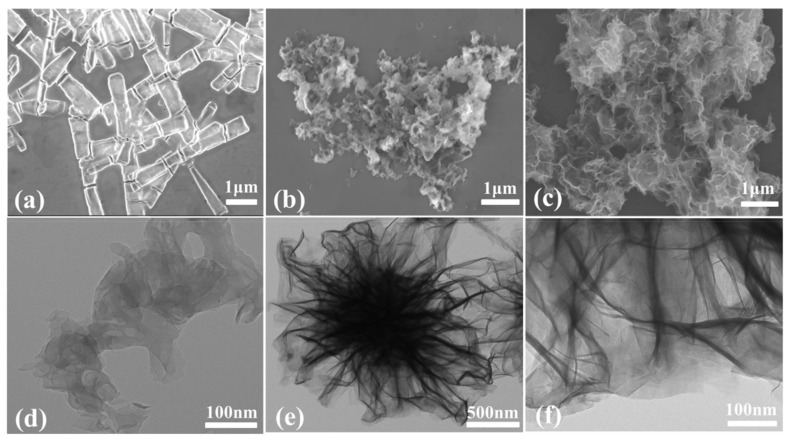
SEM images of (**a**) K_7_HNb_6_O_19_, (**b**) g-C_3_N_4_ and (**c**) Nb–CN-0.4. TEM images of (**d**) g-C_3_N_4_, (**e**) Nb–CN-0.4 and (**f**) Nb–CN-0.4.

**Figure 5:**
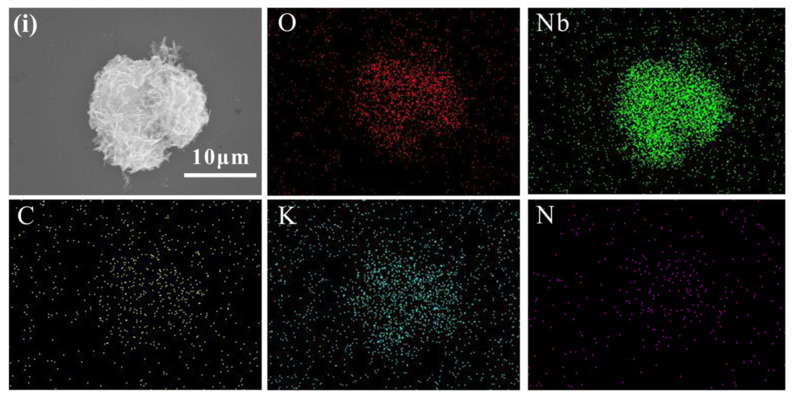
EDS element mapping images for O, Nb, C, K and N of (**i**) Nb–CN-0.4.

The morphology and structures of photocatalysts K_7_HNb_6_O_19_, g-C_3_N_4_ and Nb–CN-0.4 composite were investigated by SEM, TEM and mapping techniques. It is generally known that the Lindqvist type K_7_HNb_6_O_19_ is made up of six co-planer Nb–O octahedrons to form a super octahedron, as shown in Figure S1, leading to a highly symmetric compact structure [39]. Figure 4a and Figure S2 reveal that the pure K_7_HNb_6_O_19_ showed a transparent rodlike morphology. The pure g-C_3_N_4_ obtained by annealing urea exhibited a large piled sheet shape (Figure 4b), which was caused by thermal polycondensation of urea [40]. The SEM image of Nb–CN-0.4 composite (Figure 4c) presented a thin nanosheet morphology, just like that of the pure g-C_3_N_4_ but with better dispersion. However, K_7_HNb_6_O_19_ couldn’t be observed in the SEM image, maybe due to its highly uniform distribution caused by the good solubility of K_7_HNb_6_O_19_ under hydrothermal conditions. To further confirm the existence of K_7_HNb_6_O_19_, element mapping was performed. As shown in Figure 5, C, O, Nb, K and N elementals were distributed uniformly, further proving the formation of the heterogeneous structure containing K_7_HNb_6_O_19_ and g-C_3_N_4_. Exposing the individual components (pristine g-C_3_N_4_ and K_7_HNb_6_O_19_) to the hydrothermal condition is of importance in order to verify the reason causing morphology changes of the achieved composite catalysts in the hydrothermal process. Therefore, we treated the individual component K_7_HNb_6_O_19_ at 180 °C for 12 h, and found it was still a clear and transparent aqueous solution. After evaporating, the obtained crystals showed an identical IR to that of the freshly prepared K_7_HNb_6_O_19_ (Figure S3), indicating that K_7_HNb_6_O_19_ can exist stably under the hydrothermal condition. For the pristine g-C_3_N_4_, as seen in Figure S4, the hydrothermal treatment made its morphology change from an original sheet shape to the extremely irregular rodlike shape. Based on the comparison of the morphology of g-C_3_N_4_ before and after hydrothermal treatment, it can be concluded that the presence of K_7_HNb_6_O_19_ could positively induce the morphology change of the obtained composite, which is consistent with our previous reports [35,36]. The results of HRTEM measurement showed that the introduction of K_7_HNb_6_O_19_ made the Nb–CN-0.4 composite possess a more regular ultrathin nanosheet structure compared to that of the pure g-C_3_N_4_ (Figure 4d–f). Ultimately, a number of nanosheets stacked together to further form a flower-like shape unit of the composite (Figure 4f). For the photocatalyst Nb–CN-0.4 composed of ultrathin nanosheets, an improved charge transfer efficiency could be expected compared with pure K_7_HNb_6_O_19_ [41], which is consistent with good photocatalytic performance.

**Figure 6:**
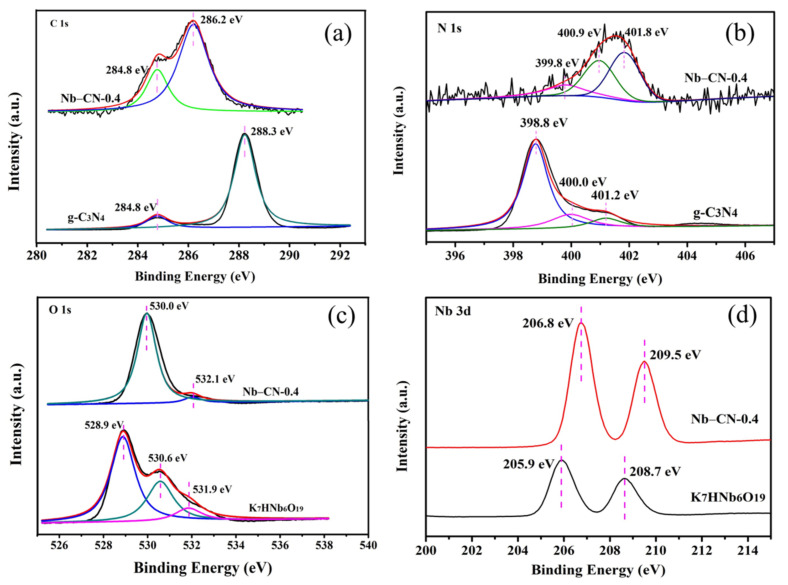
High resolution XPS spectra of K_7_HNb_6_O_19_, Nb–CN-0.4 composite and g-C_3_N_4_: (**a**) C 1s; (**b**) N 1s; (**c**) O 1s; (**d**) Nb 3d.

The surface chemical status of K_7_HNb_6_O_19_, g-C_3_N_4_ and Nb–CN-0.4 composite was studied by X-ray photoelectron spectroscopy (XPS). As shown in Figure S5, the full XPS spectra clearly show the existence of C, Nb, N, K and O elements in the composite sample, indicating that the hybrid material had all the characteristic elements of K_7_HNb_6_O_19_ and g-C_3_N_4_. In the C 1s spectrum (Figure 6A), the main characteristic peak centered at 284.8 eV could be attributed to that of sp^2^ C–C or C=C bonds, which derived from the amorphous reference carbon on the surface. For the pure g-C_3_N_4_, the peak centered at 288.3 eV corresponded to the sp^2^ carbon atoms bonded to N (N=C–(N)_2_) in the graphitic structure [42,43]. It should be noted that the C 1s peak centered at 286.2 eV in Nb–CN-0.4 showed a distinct shift toward the lower band energy direction after hydrothermal treatment compared with the pure g-C_3_N_4_, indicating the presence of strong interactions between the terminal oxygen atom of Nb=O or the bridge oxygen of Nb–O–Nb from K_7_HNb_6_O_19_ and the N=C–(N)_2_ group from g-C_3_N_4_. In addition, the strong electronic pull of the Linqvist anions might also have affected the position of the C 1s peak [30]. The three N 1s peaks in Nb–CN-0.4 composite were observed at the positions 399.8, 400.9 and 401.8 eV, with the intensity slightly greater than those of g-C_3_N_4_, corresponding to the peaks of sp^2^ N atoms involved in tris-triazine rings (C=C–N), tertiary nitrogen (N–(C)_3_) and N atoms bonded with H atoms (C–N–H) in the aromatic rings, respectively [44]. It can be seen from Figure 6c that three peaks, centered at 528.9, 530.6 and 531.9 eV, were observed on the K_7_HNb_6_O_19_ sample, which were assigned to Nb–O–Nb, Nb–O–H and adsorbed water, respectively [24]. After hydrothermal treatment, the O 1s in Nb–CN-0.4 had two main peaks, of which the peak at 532.1 eV was adsorbed water, while the other one resulted from the binding energy of different oxygen-containing species. Compared with the pure K_7_HNb_6_O_19_, the binding energy peaks located at 206.8 and 209.5 eV corresponded to Nb 3d_5/2_ and 3d_3/2_ in the Nb–CN-0.4 composite, which indicates that Nb is in its highest oxidation state (+5 valence state) [24] (Figure 6d), and the shift of Nd 3d binding energy further confirmed that there was an interaction between the Lindqvist unit of the K_7_HNb_6_O_19_ sample and g-C_3_N_4_ sample. Figure S6 shows that K 2p binding energies did not change significantly in K_7_HNb_6_O_19_ and the composite, except for their intensities.

The BET tests of K_7_HNb_6_O_19_, g-C_3_N_4_ and Nb–CN-0.4 composite were carried out, and the N_2_ adsorption–desorption isotherms are shown in Figure S7. The calculated specific surface areas of K_7_HNb_6_O_19_ and g-C_3_N_4_ were about 3.15 m^2^ g^−1^ and 52.13 m^2^ g^−1^, respectively, while that of Nb–CN-0.4 composite increased to 55.34 m^2^ g^−1^ after the introduction of g-C_3_N_4_. The larger specific surface area is conducive to the enhancement of the photocatalytic activity [45], which would promote more water molecule adsorption on the surface of the catalysts. Meanwhile, it is also beneficial for the increase of catalytic active sites.

### 3.2. Optical and Electric Properties

**Figure 7:**
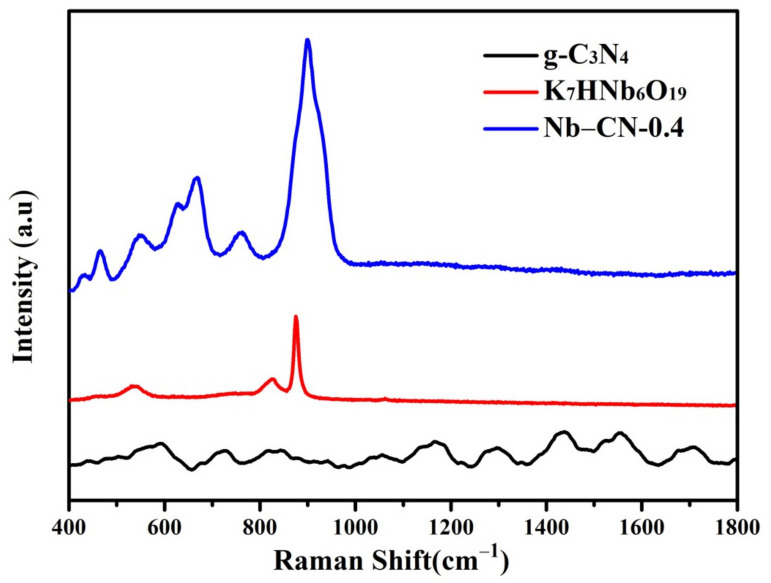
Raman spectra of K_7_HNb_6_O_19_, g-C_3_N_4_ and Nb–CN-0.4 composite.

Raman spectroscopy of K_7_HNb_6_O_19_, g-C_3_N_4_ and Nb–CN-0.4 composite are depicted in Figure 7. There were two obvious peaks at 759 and 901 cm^−1^ for Nb–CN-0.4 composite, which were attributed to the terminal oxygen and bridging oxygen in K_7_HNb_6_O_19_ [46]. The broad band in 400–700 cm^−1^ was attributed to g-C_3_N_4_ in Nb–CN-0.4 composite. As for the pure g-C_3_N_4_, the Raman peaks located at 592 cm^−1^, 725 cm^−1^, 1055 cm^−1^, 1165 cm^−1^, 1296 cm^−1^, 1441 cm^−1^ and 1550 cm^−1^ were related to CN heterocyclic stretching vibration, of which the last two in the high wavenumber region were attributed to D and G bands of g-C_3_N_4_ of a typical graphitic structure, respectively [47,48]. Compared to the bare K_7_HNb_6_O_19_ and g-C_3_N_4_, the Raman peaks of Nb–CN-0.4 composite became broader, further proving the formation of the hybrid material and matching well with the results of IR and X-ray powder diffraction analysis.

**Figure 8:**
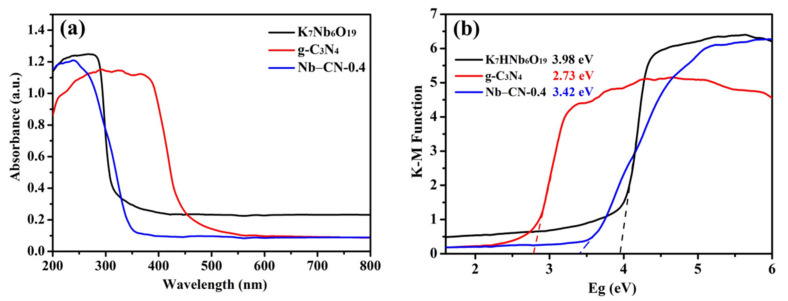
(**a**) UV−visible absorbance spectra of K_7_HNb_6_O_19_, g-C_3_N_4_ and Nb–CN-0.4 composite; (**b**) Kubelka−Munk transformed reflectance spectra of K_7_HNb_6_O_19,_ g-C_3_N_4_ and Nb–CN-0.4 composite.

The optical absorption properties of photocatalysts K_7_HNb_6_O_19_, g-C_3_N_4_ and Nb–CN-*X* composites were characterized by UV-vis DRS (Figure S8). As shown in Figure S6 and Figure 8a, K_7_HNb_6_O_19_ only absorbed ultraviolet light, and the absorption edge was about 310 nm, while all the composites showed a slightly broader absorption after introducing C_3_N_4_, which was undoubtedly attributed to the contribution of g-C_3_N_4_ with the absorption band edge around 450 nm [49]. The band gap values of as-prepared photocatalysts were calculated according to the equation of (*α*h*υ*)^2^ = A(h*υ* − *E*_g_), where *α*, h, *υ* and A represent the absorption coefficient, Plank constant, light frequency and a constant, respectively [50]. As described in Figure 8b, the band gap value *E*_g_ of the pure K_7_HNb_6_O_19_ and g-C_3_N_4_ were about 3.98 and 2.73 eV, respectively, which were consistent with literature reports [29,51]. The *E*_g_ value of Nb–CN-0.4 was about 3.42 eV, slightly narrower than that of K_7_HNb_6_O_19_, indicating the presented composite is an ultraviolet light photocatalyst without activity under visible light irradiation.

To study the separation efficiency of the photogenerated carriers of the samples, photoluminescence spectroscopies (PL) of g-C_3_N_4_ and Nb–CN-0.4 composite were performed. The fluorescence intensity of each sample was studied at the same excitation wavelength of 300 nm. Generally, a lower PL intensity indicates a lower recombination rate of the photoexcited electrons and holes, meaning a higher photocatalytic activity [52]. As shown in Figure S9, the introduction of K_7_HNb_6_O_19_ can partially quench the fluorescence of g-C_3_N_4_, which means that the introduction of K_7_HNb_6_O_19_ promotes the separation of photogenerated carriers, which is consistent with its good photocatalytic hydrogen production activity.

**Figure 9:**
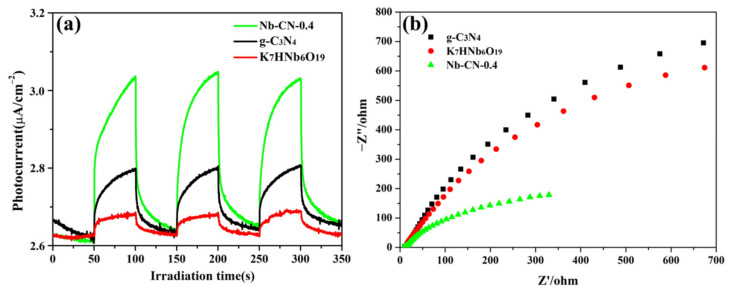
(**a**) Photocurrent response of K_7_HNb_6_O_19_, g-C_3_N_4_ and Nb–CN-0.4 composite. (**b**) Electrochemical impedance spectroscopy of g-C_3_N_4_, K_7_HNb_6_O_19_ and Nb–CN-0.4 composite.

The photocurrent transient response experiment (Figure 9a) showed that the Nb–CN-0.4 had the highest photocurrent intensity, much higher than the original g-C_3_N_4_ and K_7_HNb_6_O_19_, implying that Nb–CN-0.4 had the faster transfer efficiency and more efficient separation of photogenerated charges [53]. Moreover, the electrochemical impedance spectroscopy Nyquist plot was used to analyze the charge transfer rate (Figure 9b). The smallest arc radius of Nb–CN-0.4 under the same test conditions implied that it had the highest separation and migration rate of the photo-generated carriers [54], which was in keeping with the results of photocurrent response and photocatalytic performance studies.

### 3.3. Photocatalytic H_2_ Production

**Figure 10:**
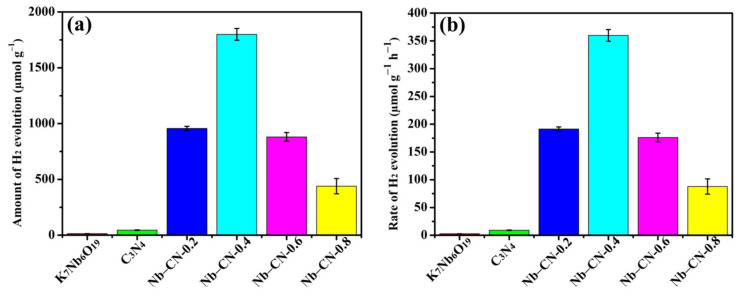
The amount (**a**) and rate (**b**) of H_2_ evolution with 5 h of illumination on pure K_7_HNb_6_O_19_, g-C_3_N_4_ and Nb–CN-*X* composites.

The photocatalytic performance of hydrogen production of K_7_HNb_6_O_19_, g-C_3_N_4_ and Nb–CN-X composites was tested using methanol (MeOH) as a sacrificial agent under a 300 W Xenon Lamp without a co-catalyst. Herein, methanol was selected as the sacrificial agent because it could act mainly and preferentially as a hole scavenger to decrease the recombination rate of photogenerated charge carriers for enhanced photocatalytic H_2_ evolution efficiency, similar to our previous work [35,55]. Typically, 50 mg photocatalyst powder was added into the mixed solution of 40 mL deionized water and 10 mL methanol, and then stirred and sonicated for 15 min to ensure the uniform dispersion of the catalyst; the concentration of the solid catalyst was 1mg/mL. As shown in Figure 10, the average hydrogen evolution rates were 2.72 µmol g^−1^ h^−1^ for K_7_HNb_6_O_19_ and 9.05 µmol g^−1^ h^−1^ for g-C_3_N_4_. After combining K_7_HNb_6_O_19_ with g-C_3_N_4_, Nb–CN-0.4 composite exhibited the highest hydrogen production rate, as much as 359.89 µmol g^−1^ h^−1^, approximately 133 and 40 times of the original K_7_HNb_6_O_19_ and g-C_3_N_4_, respectively. To the best of our knowledge, the maximum rate of hydrogen production reported is 207.6 μmol h^−1^ g^−1^ for such kinds of photocatalytic materials in the literature (Table S1). Therefore, Nb–CN-0.4 composite represented the binary PONb-based photocatalytic material with the highest hydrogen production rate up to now. Figure 10b showed that with the amount of g-C_3_N_4_ increasing, the H_2_ producing activity of Nb–CN-X composites increased gradually to a maximum value and then decreased. Although the coupling of K_7_HNb_6_O_19_ and g-C_3_N_4_ could effectively increase the hydrogen production efficiency and significantly improve the photocatalytic performance, the excessive g-C_3_N_4_ would bring about a shielding effect and introduce more recombination centers, which was not conducive to the charge separation, leading to a low performance of photocatalytic hydrogen production.

**Figure 11:**
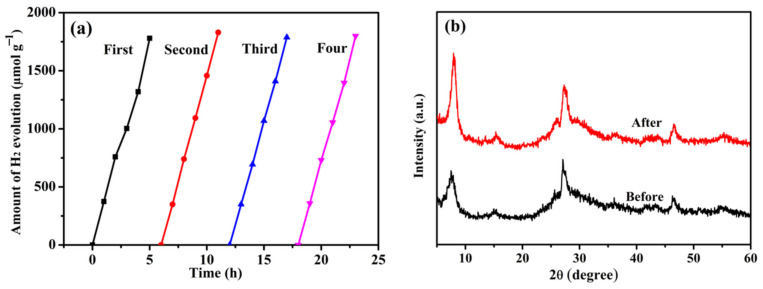
(**a**) Nb–CN-0.4 composite hydrogen production cycle effect; (**b**) XRD patterns of Nb–CN-0.4 composite before and after four cycle tests.

To verify the stability of the prepared photocatalyst, the recyclability performance of the optimal sample of Nb–CN-0.4 composite was evaluated under the same condition. As shown in Figure 11a, after four recycling experiments, the hydrogen evolution rate did not decrease obviously, indicative of a high stability of the sample. Moreover, the crystallinity of the catalyst was retained well after four rounds of recycling, which could be drawn from the XRD pattern in Figure 11b, further proving the good structural and catalytic stability of the prepared composite.

### 3.4. Investigation of Photocatalytic Mechanism

**Figure 12:**
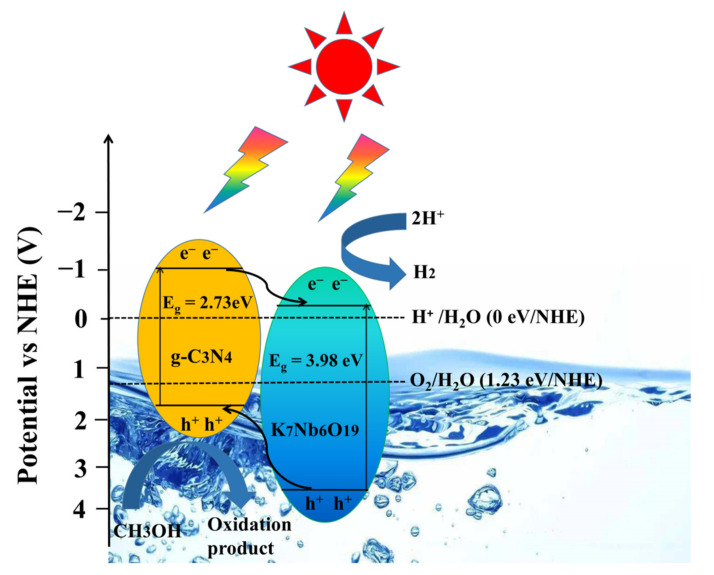
The possible photocatalytic mechanism of type-II heterojunction photocatalyst Nb–CN-0.4 composite hydrogen production.

The valence band XPS spectra test was carried out to study the photocatalytic mechanism (Figure S10). The estimated valence band (VB) positions of K_7_HNb_6_O_19_ and g-C_3_N_4_ were 3.61 and 1.67 eV vs. NHE, respectively, which were consistent with the reported values [36]. The conductance band (CB) positions (E_CB_) of K_7_HNb_6_O_19_ and g-C_3_N_4_ were calculated to be −0.37 and −1.06 eV vs. NHE based on the empirical formula of *E*_g_ = *E*_VB_ − *E*_CB_. Based on the above analysis and the photocatalytic experimental results, we proposed a possible mechanism of photocatalytic hydrogen production, as seen in Figure 12. On the basis of the definition of a heterojunction [56], a type-II heterojunction photocatalytic system could be formed between K_7_HNb_6_O_19_ and g-C_3_N_4_ due to a staggered band gap. Under the full spectral irradiation, the electrons were excited from VB to CB of both g-C_3_N_4_ and K_7_HNb_6_O_19_. Because the CB potential of g-C_3_N_4_ was lower than that of K_7_HNb_6_O_19_, the photo-induced electrons on the CB of g-C_3_N_4_ could migrate more easily to the CB of K_7_HNb_6_O_19_, while the photo-induced h^+^ were concentrated on the VB of g-C_3_N_4_ simultaneously. For the photocatalytic H_2_ production, the electrons accumulated on the CB of K_7_HNb_6_O_19_ could be captured by H^+^ to produce H_2_, and the holes in the VB of g-C_3_N_4_ were consumed by the sacrificial agent CH_3_OH. The mechanism of photocatalytic hydrogen evolution is shown in Figure 12, and the pathways can be illustrated as follows:

Nb–CN-*X* + h*υ* → Nb–CN-*X* + e^−^ + h^+^ (1)

e^−^ + 2H^+^ → H_2_ (2)

h^+^ + CH_3_OH → CH_3_OH^+^ (3)

## 4. Conclusions

In summary, a series of binary type-II heterojunction Nb–CN-*X* composites were successfully prepared via a one-step hydrothermal method. The obtained optimal photocatalyst Nb–CN-0.4 composite displayed superior photocatalytic H_2_ generation activity, with a H_2_ generation rate of 359.89 µmol g^−1^ h^−1^. The binary type-II heterojunction photocatalyst containing g-C_3_N_4_ and polyoxoniobate (PONb, K_7_HNb_6_O_19_) with excellent H_2_ production activity under ultraviolet light driving was synthesized by decorating via a facile hydrothermal method for the first time and without any co-catalyst, which was approximately 133 and 40 times than that of bare K_7_HNb_6_O_19_ and g-C_3_N_4_. The enhanced photocatalytic hydrogen evolution was ascribed to a good heterojunction formed between K_7_HNb_6_O_19_ and g-C_3_N_4_, which significantly accelerated the charge carries transfer rate on the interface and improved the separation of photogenerated electrons and holes, as demonstrated by the result of photochemical tests. It is worth mentioning that Nb–CN-*X* composites are the first example of binary type-II heterojunction K_7_HNb_6_O_19_/g-C_3_N_4_ photocatalyst to H_2_ evolution. This work could provide reference for the preparation and modification of PONb-based photocatalysts with low cost and high performance, as well as their further application in clean energy.

## Data Availability

Data is contained within the article or Supplementary Materials.

## Supplementary Materials

The following supporting information can be downloaded at: https://www.mdpi.com/article/10.3390/nano12050849/s1, Figure S1: Configuration of Lindqvist type K_7_HNb_6_O_19_, green sphere is Nb, red sphere is oxygen. Figure S2: SEM of K_7_HNb_6_O_19_. Figure S3: IR of K_7_HNb_6_O_19_ before and after hydrothermal. Figure S4: SEM of individual component g-C_3_N_4_ (a) Before hydrothermal treatment (b) After hydrothermal treatment. Figure S5: XPS of g-C_3_N_4_, K_7_HNb_6_O_19_ and Nb–CN-0.4 composite in the survey spectra. Figure S6: High resolution K 2p XPS of the pure K_7_HNb_6_O_19_ and Nb–CN-0.4 composite. Figure S7: N_2_ adsorption-desorption isotherms of K_7_HNb_6_O_19_ and Nb–CN-0.4 composite. Figure S8: UV–vis DRS spectra of Nb–CN-*X* composites. Figure S9: PL spectra of g-C_3_N_4_ and Nb–CN-0.4 composite. Figure S10: Valence band XPS spectra of K_7_HNb_6_O_19_, g-C_3_N_4_. Table S1: Hydrogen production activities of the binary polyoxoniobates materials reported. References [57,58,59,60,61] are cited in the Supplementary Materials.
