# Supplementary material for: Binary Type-II Heterojunction K7HNb6O19/g-C3N4: An Effective Photocatalyst for Hydrogen Evolution without a Co-Catalyst
Source: Nanomaterials (Basel). 2022 Mar 2;12(5):849. doi: 10.3390/nano12050849 (PMC8912307; doi:10.3390/nano12050849)
Supplement: Supplementary file 1 [file nanomaterials-12-00849-s001.zip › nanomaterials-1593173-supplementary.pdf]

Supplementary Materials

Binary Type-II Heterojunction $K_7HNb_6O_{19}$/g-C_3N_4: An Effective Photocatalyst for Hydrogen Evolution without a Co-Catalyst

Qi Song †, Shiliang Heng †, Wenbin Wang, Huili Guo, Haiyan Li and Dongbin Dang *

Henan Key Laboratory of Polyoxometalate Chemistry, College of Chemistry and Chemical Engineering, Henan University, Kaifeng 475004, China; songqi1666@163.com (Q.S.); hengshiliangecnu@163.com (S.H.); wwenbin2022@163.com (W.W.); ghlhenu@163.com (H.G.); lihaiyan@henu.edu.cn (H.L.)

* Correspondence: dangdb@henu.edu.cn

† These authors contributed equally to this work.

1. Experimental

1.1. Analytic Methods and Characterization of Samples

X-ray diffractometer (XRD, Bruker D8 Advance diffractometer, Germany) equipped with Cu Kα radiation source was used to study crystalline phase of catalysts. The morphology of catalysts was obtained on Field emission scanning electron microscope (FESEM, JSM-7610F, Japan) equipped with an energy-dispersive X-ray spectroscopy and High-resolution transmission electron microscopy (HRTEM, JEM-2100, Japan) operating at 200 kV. Fourier transform infrared spectra were recorded on a Fourier transform infrared spectrometer (VERTEX 70, Germany) in the range of 4000–400 cm^{-1} at KBr phase. The surface area of the sample was recorded on Brunauer-Emmett-Teller nitrogen absorption method (BET, BELSORP-Max II, Japan) with N_2 adsorption-desorption isotherms at 77 K. X-ray photoelectron spectroscopic (XPS, ESCALAB 250 Xi, USA) with Al-Kα as the X-ray source was conducted to analyze the surface electronic state and valence band potentials of the photocatalyst. Raman spectroscopy was carried out on a DXR spectrometer (Renishaw inVia, UK) using the 532 nm laser. UV-vis diffuse reflectance spectra (DRS, Shimadzu, UV-2600, Japan) of the samples were achieved using the $BaSO_4$ powder as the reference. Photoluminescence spectra (PL, TU-1900, China) were recorded at the exaction wavelength of 300 nm. Photoluminescence spectroscopy (PL) is performed in solid-state.

1.2. Photocatalytic Hydrogen Evolution Experiment

The photocatalytic water splitting to product H_2 experiment was completed using an online photocatalytic system (CEL-PF300-T8, AULTT, China Co, Ltd). The detection device is composed of a closed glass instrument and a vacuum pumping circulation system connected with gas chromatography. The carrier gas is high-purity argon, and the automatic sampler is collected every 60 min. 5A column is used as the gas separation column for analysis through the thermal conductivity detector (TCD) system of the online gas chromatograph. 50 mg photocatalyst powder was added into mixed solution of 40 mL deionized water and 10mL methanol as sacrificial agent, stirred and sonicated for 15 min to ensure the uniform dispersion of the catalyst. Connect the reactor to the glass instrument, and vacuumize the mixture for 30 min before irradiation to completely remove the air in the reaction system. The 300 W xenon lamp (Beijing Perfect Light Technology Co. Ltd., China) was used as the simulated sunlight source, and circulating condensed water was opened in the reaction process to ensure that the temperature in the whole reaction process was kept at 6 °C. In order to test the stability of the catalyst, the photocatalytic H_2 production cycle experiment was carried out. The experiment process was the same as the above operation, except for after each cycle, the system was again evacuated for the next cycle experiment.

1.3. Photoelectrochemical Measurement

Electrochemical measurements were analyzed using the CHI-660 electrochemical workstation with 0.2 M Na_2SO_4 aqueous solutionas the electrolyte in a typical three-electrode mode. The working electrode was prepared following the procedure: 5.0 mg Nb–CN-0.4 composite powders were mixed with 300.0 μL ethanol/Nafion (v/v = 19/1) under sonication for 30 min. 30.0 mL as-prepared slurry was cast onto the surface of an FTO substrate (1.0 cm × 1.0 cm) as thin-film via drop-casting method and then dried in air for 30 min. Finally, the working electrode was successfully fabricated after this process was repeated several times to obtain a smooth and uniform coating. A Pt electrode and a saturated calomel electrode (SCE) were used for the counter electrode and reference electrode, respectively. Electrochemical impedance spectroscopy (EIS) measurements were carried out in dark, with frequencies of 1000 Hz. The transient photocurrent responses were measured upon the illumination of a 300 W Xenon lamp (Beijing Perfect Light Technology Co., Ltd.) as the light source. The 0.2 M Na_2SO_4 aqueous solution was used as a supporting electrolyte and high-purity N_2 was passed into the solution for at least 20 min to remove oxygen before test.

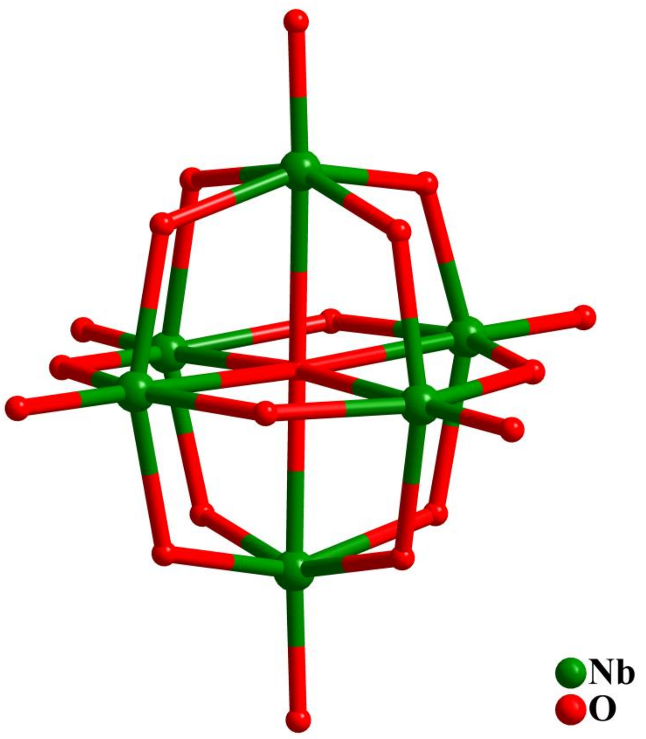

Figure S1. Configuration of Lindqvist type $K_7HNb_6O_{19}$, green sphere is Nb, red sphere is oxygen

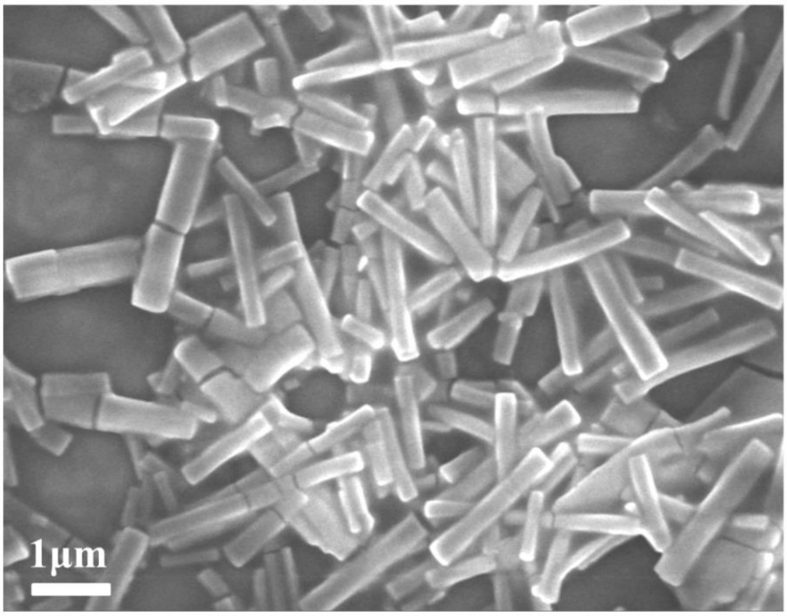

Figure S2. SEM of $K_7HNb_6O_{19}$.

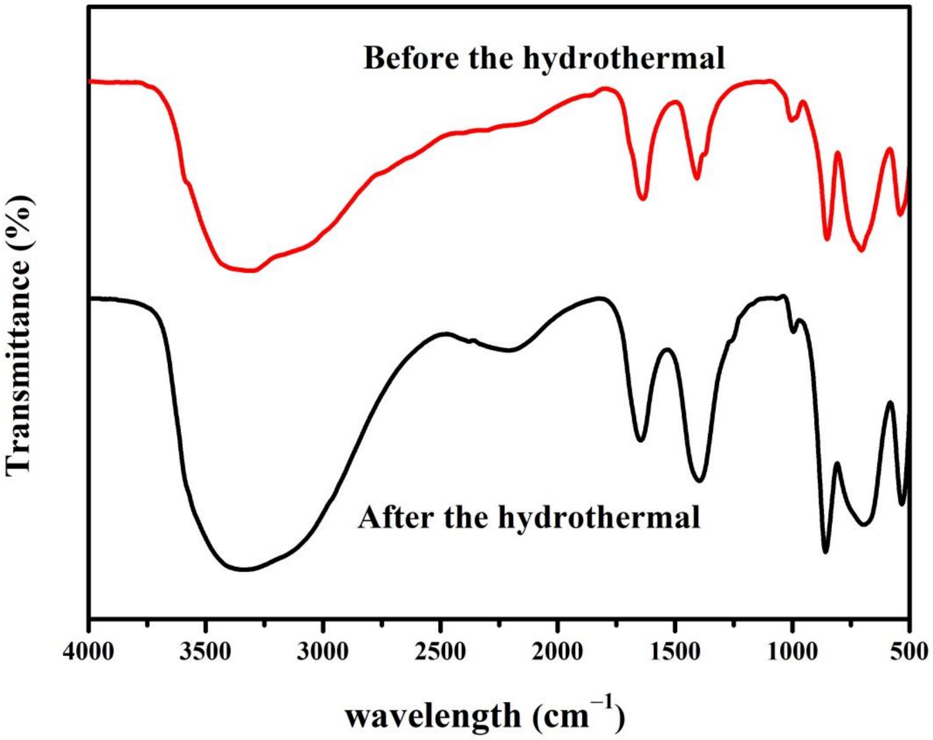

Figure S3. IR of $K_7HNb_6O_{19}$ before and after hydrothermal treatment.

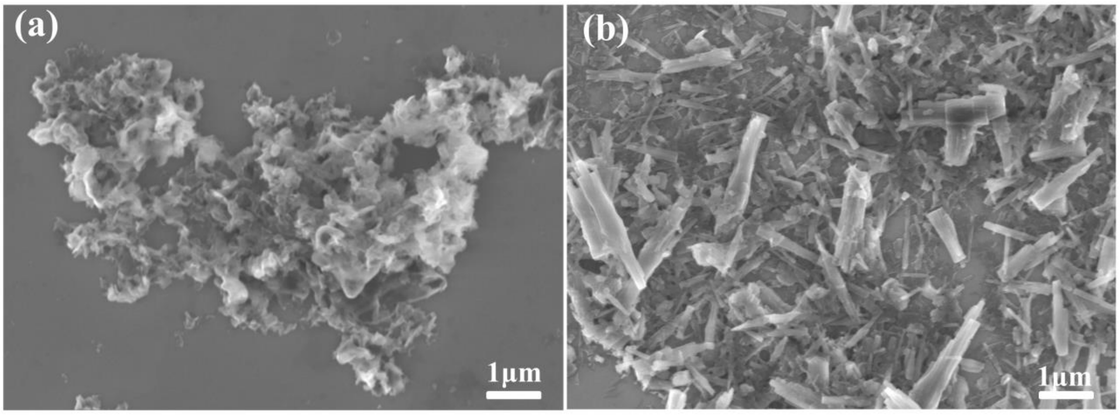

Figure S4. SEM of individual component g-C_3N_4 (a) Before hydrothermal treatment (b) After hydrothermal treatment.

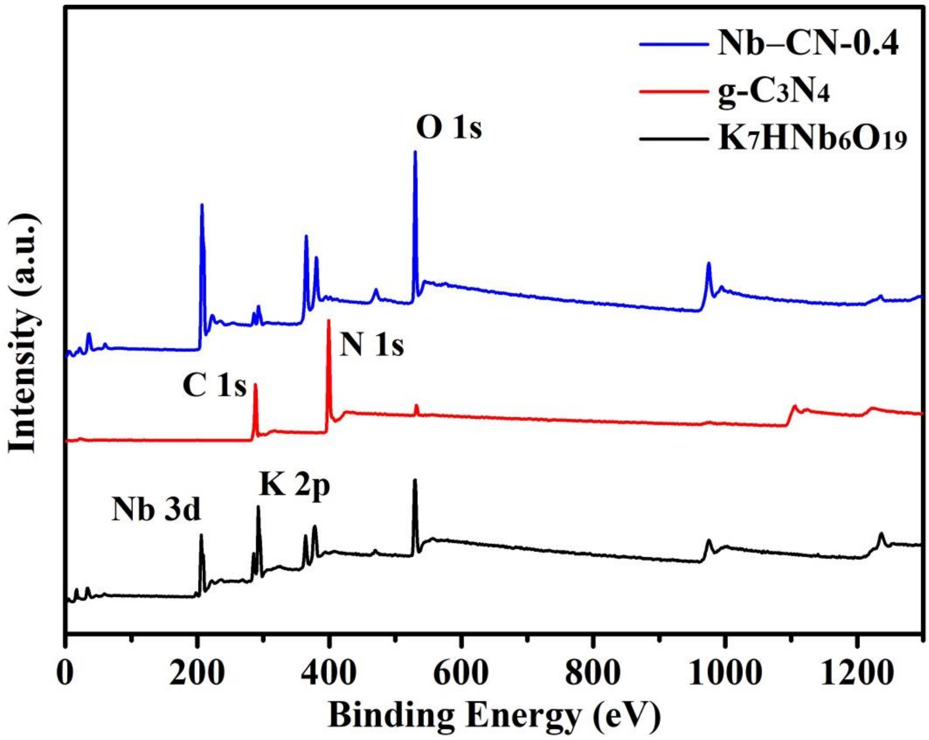

Figure S5. XPS of Nb−CN-0.4 composite in the survey spectra.

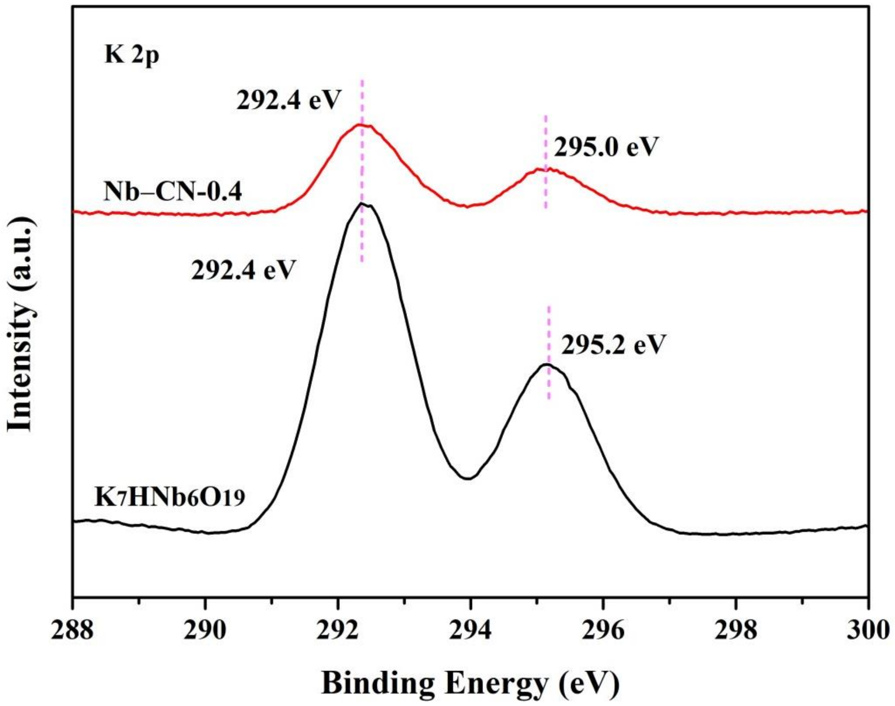

Figure S6. High resolution K 2p XPS of the pure $K_7HNb_6O_{19}$ and Nb−CN-0.4 composite.

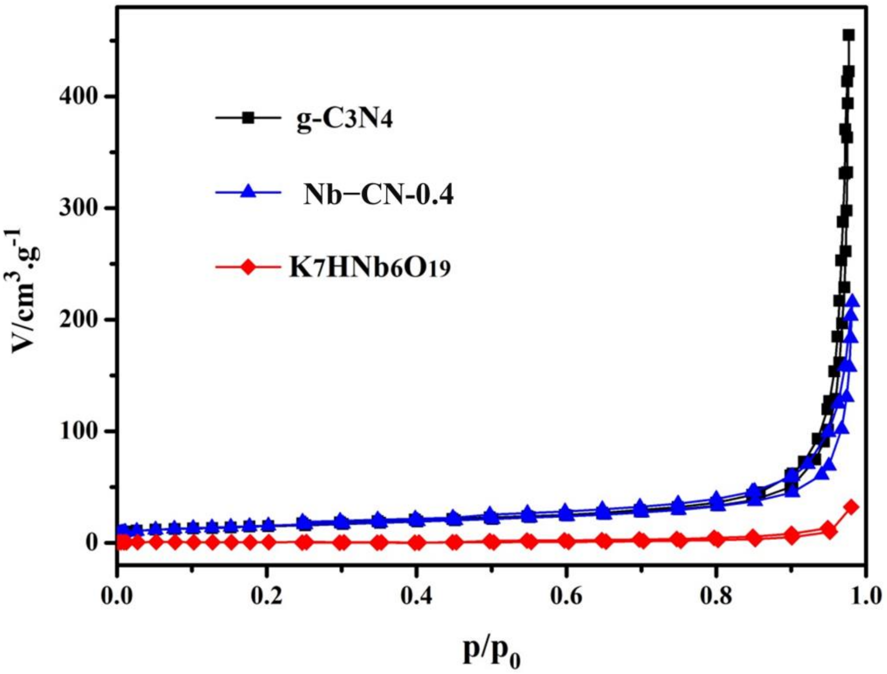

Figure S7. N_2 adsorption-desorption isotherms of $K_7HNb_6O_{19}$, g-C_3N_4 and Nb−CN-0.4 composite.

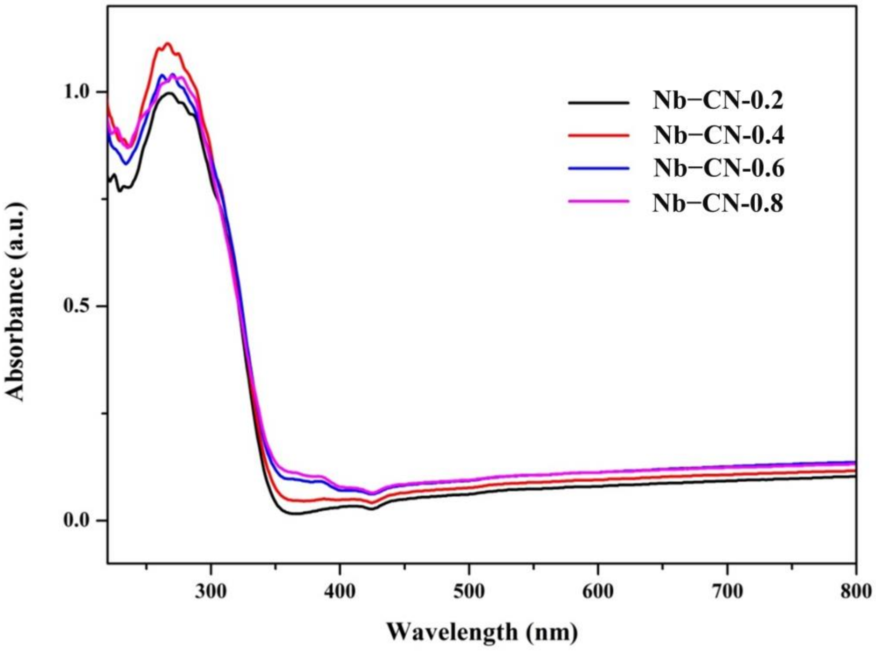

Figure S8. UV–vis DRS spectra of Nb−CN-*X* composites.

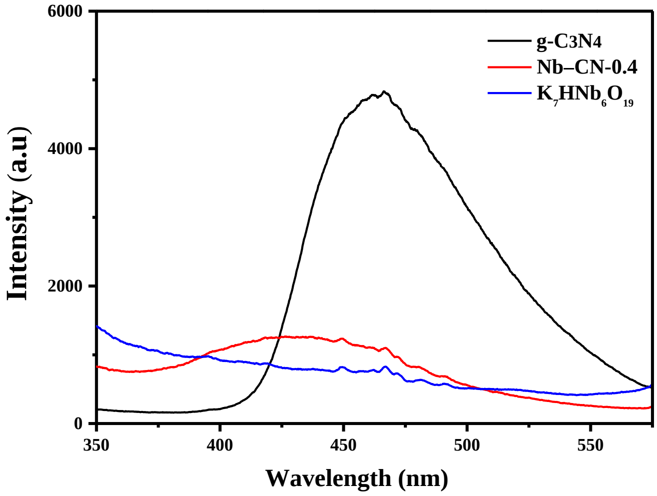

Figure S9. PL spectra of $K_7HNb_6O_{19}$, g-C_3N_4 and Nb−CN-0.4 composite.

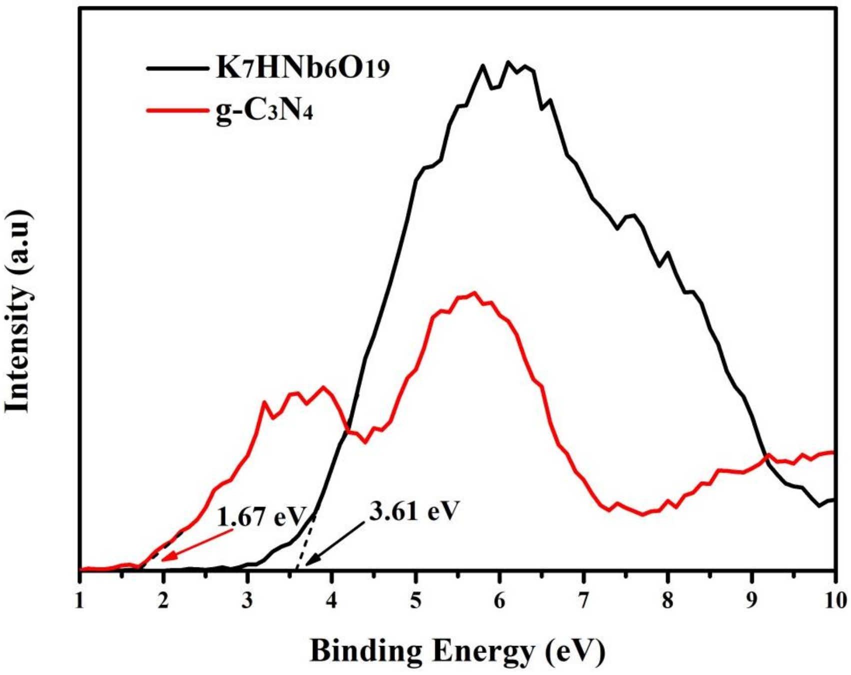

Figure S10. Valence band XPS spectra of $K_7HNb_6O_{19}$, g-C_3N_4.

Table S1. Hydrogen production activities of the binary polyoxoniobates materials reported.

Photocatalyst	Light	Co-Catalyst	Rate	Temperature/ Catalyst concentrations	Sacrificial Agents	Refs
$[Cu(en)_2]_{11}K_4Na_2[KNb_{24}O_{72}H_9]_2$	500 W Xe (λ > 400 nm)	0.1001 g $[Co^{III}(dmgH)_2pyCl]$	13.2 μmol h^{-1} g^{-1}	-/1 mg ml^{-1}	10% TEA	[57]
$\{Cs_{2.5}H_{1.5}Na_2\{As_2Nb_4(O_2)_4O_{14}\}$	300 W Xe	1.0% H_4PtCl_6	102.3 μmol h^{-1} g^{-1}	-/1 mg ml^{-1}	20% CH_3OH	[58]
$[Co_{14}(OH)_{16}(H_2O)_8Nb_{36}O_{106}]$	300 W Xe	0.5% H_4PtCl_6	44 μmol h^{-1} g^{-1}	-/1 mg ml^{-1}	20% CH_3OH	[59]
$[Cu(en)_2]_4[PNb_{12}O_{40}(VO)_6](OH)_5$	300 W Xe	0.75% H_4PtCl_6	10.31 μmol h^{-1} g^{-1}	-/1 mg ml^{-1}	20% CH_3OH	[60]
$[Cu(enMe)_2]_4[PNb_{12}O_{40}(VO)_6](OH)_5$	300 W Xe	0.75% H_4PtCl_6	10.45 μmol h^{-1} g^{-1}	-/1 mg ml^{-1}	20% CH_3OH	[60]
$[Co(pn)_2]_4[HPNb_{10}V^{IV}2O40(V^{IV}O)_4]$	300 W Xe	1.0% H_4PtCl_6	19.25 μmol h^{-1} g^{-1}	-/1 mg ml^{-1}	10% CH_3OH	[61]
$[Co(pn)_2]_5[PNb_{12}O_{40}(V^{IV}O)_6](OH)_7$	300 W Xe	1.0% H_4PtCl_6	29.25 μmol h^{-1} g^{-1}	-/1 mg ml^{-1}	10% CH_3OH	[61]
$K_4Nb_6O_{17}$/g-C_3N_4	300 W Xe (λ > 420 nm)	2 wt.% Pt	16.91 μmol h^{-1} g^{-1}	-/0.5 mg ml^{-1}	10% TEOA	[35]
$K_7HNb_6O_{19}$/PPy-RGO	300 W Xe	-	207.6 μmol h^{-1} g^{-1}	5 °C/1mg ml^{-1}	20% CH_3OH	[31]
$K_7HNb_6O_{19}$/g-C_3N_4	300 W Xe	-	359.8 μmol h^{-1} g^{-1}	6 °C/1mg ml^{-1}	20% CH_3OH	This study
